# Immunogenicity and Safety of a Chemically Synthesized Divalent Group A Streptococcal Vaccine

**DOI:** 10.1155/2018/4702152

**Published:** 2018-02-28

**Authors:** Yongxiang Wu, Suhua Li, Yanting Luo, Yunyue Zhao, Jiarui Wang, Ruimin Dong, Xujing Xie, Jieming Zhu, Jinlai Liu

**Affiliations:** Department of Cardiology, The Third Affiliated Hospital, Sun Yat-sen University, Guangzhou 510630, China

## Abstract

**Background:**

Group A streptococcus (GAS) infections and poststreptococcal sequelae remain a health problem worldwide, which necessitates searching for an effective vaccine, while no licensed GAS vaccine is available. We have developed a divalent peptide vaccine composed of 84 amino acids to cover the main GAS serotypes (M1 and M12 streptococci) in China, and herein, we aimed to evaluate immunogenicity and safety of this vaccine.

**Methods:**

Mice were immunized with the vaccine. ELISA, indirect bactericidal test, and immunofluorescent assay were used to study immunogenicity. GAS challenge assay was used to test the protective effect. Safety was tested by histopathological analysis.

**Results:**

Immunized group mice (*n*=16) developed higher titer antibody after immunization than nonimmunized group mice (*n*=16) did. This antibody can deposit on the surface of GAS and promote killing of GAS, resulting in 93.1% decrease of M1 GAS and 89.5% of M12 GAS. When challenged with M1 and M12 streptococci, immunized group mice had a higher survival rate (87.5% and 75%) than nonimmunized group mice (37.5% and 25%). No autoimmune reactions were detected on organs of mice.

**Conclusion:**

The results suggest that this vaccine shows fair immunogenicity and safety, which will lead our research on GAS vaccine into clinical trial.

## 1. Introduction

Group A streptococcus (GAS) is an exclusive human pathogen causing a wide spectrum of diseases varying from uncomplicated pharyngitis and pyoderma to lethal invasive diseases, such as bacteremia, streptococcal toxic shock syndrome, and necrotizing fasciitis. GAS infection also results in several autoimmune sequelae including rheumatic fever, rheumatic heart disease, and poststreptococcal glomerulonephritis [1]. These diseases affect many indigenous communities of developed countries and many developing countries where health care and antibiotic treatment are poor. However, there is a reemergence of GAS infection in industrialized countries where the incidence of GAS disease has declined in the past decades [2, 3]. GAS diseases are important causes of morbidity and mortality in the world [4], which highlight the need for a safe and effective vaccine.

Efforts to develop a GAS vaccine have lasted for decades. Most strategies focus on the M protein, a surface protein with a high changeable amino terminal and a conserved carboxyl terminal [5]. M protein is an important virulence determinant and protective antigen of GAS. Previous studies have revealed that epitopes from the amino terminal of M protein are able to elicit type-specific bactericidal antibody which has been the basis for the design of complex multivalent amino terminal vaccines including the 26-valent [6] vaccine and the 30-valent [7] vaccine, and that epitopes from carboxyl terminal are capable of evoking broad serotype antibody which provided the rationale for creating conserved carboxyl terminal M protein vaccines including J8-DT [8] and StreptInCor [9].

However, effort to develop a GAS vaccine has been hindered by the diversity of streptococcal serotypes. More than 200 emm types have been identified; the distribution of GAS emm types varies from region to region [10]. Infection with one strain does not provide immunity against another strain. The 26-valent vaccine covered 90% of the serotypes in the USA but only 39% in Africa and 24% in the Pacific region [10]. Although the 30-valent vaccine evoked cross-opsonic antibodies against 24 of 40 nonvaccine serotypes [7], with more than 200 emm types, it is far away from being effective worldwide. A vaccine covering all the serotypes is unlikely to be developed, while people in GAS endemic areas are in badly need of an effective vaccine; thus geographical region-specific vaccines that encompass the multiple N-terminal epitopes of dominant serotype GAS in a given area may be feasible.

In China, emm1 and emm12 GAS are most prevalent according to our [11] and others' [12–14] investigations. In 2011, an emm12 GAS strain caused large outbreaks of scarlet fever in Shanghai [15] and Hong Kong [15, 16], resulting in two children's death in Hong Kong. Thus, we developed a divalent 84 amino acid peptide vaccine composed of three subunits: a 35 amino acid sequence from N terminal of M1 protein (M1 peptide), a 35 amino acid sequence from N terminal of M12 protein (M12 peptide), and the J14 peptide shared by conserved C terminal of these two M proteins [17]. This vaccine was demonstrated to be able to elicit high titer opsonic antibody in mice in our previous work, but two questions remain unanswered. First, is this antibody protective against GAS challenge? Second, does this vaccine induce deleterious reaction? This paper aims to answer these questions.

## 2. Materials and Methods

### 2.1. Chemically Synthesized Peptide Vaccine, GAS Strains, and Mice

**Figure 1:**
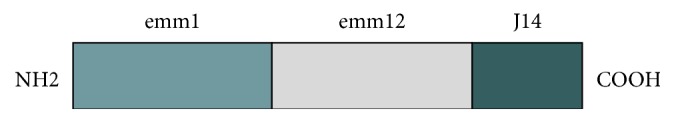
Schematic diagram of the vaccine. The vaccine is a signal peptide composed of 84 amino acids: GFANQTEVKANGDGNPREVIEDLAANNPAIQNIRLDHSDLVAEKQRLEDLGQKFERLKQRSE-LYLQQYYDASREAKKQVEKALE. The first 35 amino acid sequence (GFANQTEVKANGDGNPREVIEDLAANNPAIQNIRL) is from N terminal of M1 protein, the next 35 amino acid sequence (DHSDLVAE KQRLEDLGQKFERLKQRSELYLQQYYD) is from N terminal of M12 protein, and the last 14 amino acid sequence(ASREAKKQVEKALE) is the J14 peptide.

The amino acid sequence of the divalent peptide vaccine is GFANQTEVKANGDGNPREVIEDLAANNPAIQNIRLDHSDLVAEKQRLEDLGQKFERLKQRSELYLQQYYDASREAKKQVEKALE. The vaccine (Figure 1) and its three subunit peptides were synthesized using a 9-a-fluorenylmethoxycarbonyl (Fmoc) solid-phase strategy and purified by reverse-phase high-pressure liquid chromatography (GL Biochem, China). Specific pathogen-free 5- to 6-week-old female BALB/c mice were purchased from and maintained in a SPF animal room of Laboratory Animal Center, Sun Yat-sen University, China. The mice were housed in cages with free access to food and water. All procedures were reviewed and approved by the Animal Ethical and Welfare Committee of Sun Yat-sen University (project no. IACUC-DB-16-0407). The emm1 and emm12 GAS were isolated from human tonsil or throat samples, identified by emm gene amplification and sequencing, and preserved in our laboratory as previously described [17].

### 2.2. Immunization

Thirty-two female BALB/c mice were divided into two groups: immunized group and control group. Immunized group mice were immunized subcutaneously with 100 *μ*L of antigen-adjuvant suspension containing 20 *μ*g peptide vaccine emulsified in complete Freund's adjuvant (CFA) (Sigma). Three booster immunizations were given on day 21, day 31, and day 41 after first immunization with 20 *μ*g peptide vaccine emulsified in incomplete Freund's adjuvant (IFA) (Sigma). Control group mice received injections with the mixture of adjuvant (CFA or IFA) and PBS on the same schedule. Blood samples were obtained 1 day prior to the first immunization and 14 days after the final booster by retro-orbital puncture under light anesthesia, and then sera were collected and stored at −20°C.

### 2.3. Enzyme-Linked Immunosorbent Assay (ELISA)

Serum antibody titers were measured by ELISA with the divalent peptide as solid-phase antigens. Polyvinyl plates (CORNING) were coated with 100 *μ*L of 10 *μ*g/ml divalent peptide in 50 mM carbonate coating buffer (pH 9.6) overnight at 4°C and blocked with 5% BSA in PBS for 1 h at 37°C. The plates were then washed with 0.2% Tween-20 in PBS (PBST) for three times, and serial twofold dilutions of the sera in PBS, starting at an initial dilution of 1 : 200 to a final dilution of 1 : 2,04,800, were added to the plates (100 *μ*L/well). After 1 h at 37°C, the plates were washed with PBST for three times and incubated with horseradish peroxidase-conjugated goat antimouse IgG at a 1 : 20,000 dilution (100 *μ*L/well) at 37°C for 1 h. The plates were washed and then incubated with 3,3′,5,5′-tetramethylbenzidine (Beyotime) for 30 min at room temperature. The reaction was stopped with 50 *μ*L of 2 M H_2_SO_4_, and the absorbance was measured at 450 nm with an ELISA plate reader (BioTek Eon). Titers were defined as the reciprocal of the highest dilution of sera resulting in an optical density (OD) 2 times higher than the average OD of wells containing control group mice sera diluted at 1 : 100. The M1 peptide, the M12 peptide, and the J14 peptide were also used as ELISA solid-phase antigens to test sera antibody responses to them, respectively.

### 2.4. Indirect Bactericidal Tests

Emm1 and emm12 GAS were grown, respectively, in 5 mL of Todd–Hewitt Broth overnight at 37°C and then serially diluted to 10^5^ in PBS. For each individual mouse, 50 *μ*L of serum was mixed with 50 *μ*L of the bacterial dilution and incubated for 20 min at room temperature, and then 400 *μ*L of nonopsonic heparinized human donor blood was added. All donor blood samples had been tested to support the growth of GAS before the assay. The mixtures were incubated on shaking table at room temperature for 3 h, and 50 *μ*L of the mixtures was cultured on blood agar plate in duplicate overnight at 37°C. The number of colony-forming unit (CFU) on each plate was counted. The results were expressed as percent killing, which was calculated by using the following formula: (1−(mean CFU in the presence of immunized mouse serum)/(mean CFU in the presence of control mouse serum)) × 100.

### 2.5. Immunofluorescent Assays

Emm1 and emm12 GAS were grown, respectively, in 5 mL of Todd–Hewitt Broth overnight at 37°C, centrifuged at 3000 g for 10 min, and then resuspended in 5 mL of PBS. One hundred microliter of the bacterial suspension and 400 *μ*L of sera of mice at a 1 : 200 dilution in PBS were mixed in EP tubes and incubated at room temperature for 1 h. The mixture was then centrifuged at 3000 g for 10 min and resuspended in 400 *μ*L of PBS, which are repeated for three times to wash the bacteria. After the third centrifugation, PBS was removed and 400 *μ*L of 5% bovine serum albumin was added to resuspend the bacteria. After 1 h at room temperature, the bacterial suspension was centrifuged and washed with PBS for three times. PBS was removed, and the bacteria were resuspended in DyLight 488-conjugated goat antimouse IgG secondary antibody at a 1 : 400 dilution in PBS and incubated in dark at room temperature for 1 h. The bacterial suspension was centrifuged and washed and resuspended in 100 *μ*L of PBS. Ten microliter of the bacterial suspension was smeared across microscope slides and air-dried at room temperature. Slides were examined using a fluorescent microscope (Leica).

### 2.6. Challenge

Twenty-one days after the last immunization, 8 mice in the immunized group and 8 mice in the control group were challenged by intraperitoneal injection of 1.0 × 10^8^ CFU M1 GAS in 100 *μ*L PBS. Another 16 mice were challenged with the equivalent amount of M12 GAS. Mice in each group were observed for 15 days, and mortality was recorded to determine survival rates.

### 2.7. Histopathological Analysis

Four mice receiving a high-dose immunization and 4 mice in the control group were euthanized 6 months after the last immunization. The organs were collected, maintained in 4% formaldehyde solution, and evaluated after hematoxylin and eosin stain.

### 2.8. Statistical Analysis

Antibody titer and bacterial CFU data were analyzed by Student's *t*-test, and survival data were analyzed by log-rank Kaplan–Meier test, both using GraphPad Prism 5 software. *P* values were considered significant at <0.05.

## 3. Results

### 3.1. The Chemically Synthesized Divalent Vaccine Elicits High Titer Antibody in Mice

**Figure 2:**
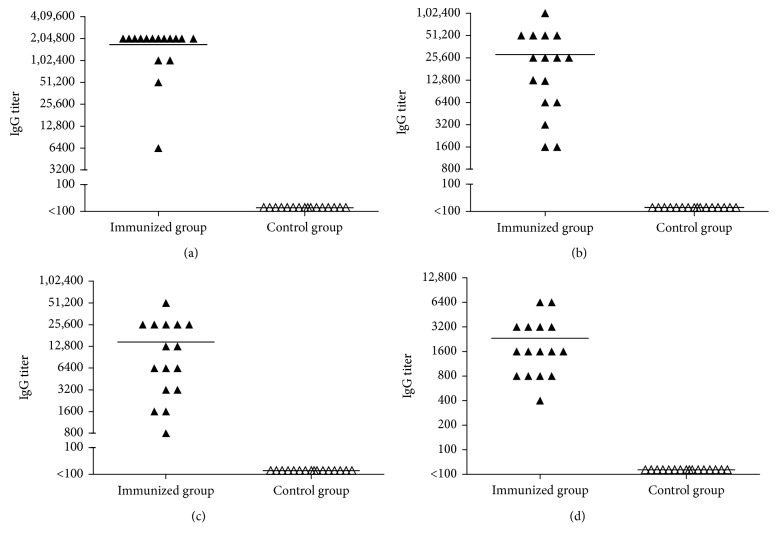
Comparison of serum IgG titers of mice. Sera were collected 14 days after the final immunization. Immunized group mice (*n*=16, black triangles) were immunized with 20 *μ*g peptide vaccine emulsified in Freund's adjuvant on days 1, 21, 31, and 41. Control group mice (*n*=16, open triangles) were immunized with PBS and Freund's adjuvant on days 1, 21, 31, and 41. Horizontal bar indicates the average antibody responses to the (a) vaccine; (b) M1 peptide; (c) M12 peptide; and (d) J14 peptide. *P* < 0.05, respectively.

ELISA assay was performed to evaluate immunogenicity of the vaccine. As shown in Figure 2, all the mice of the immunized group developed high titer antibody after three immunizing boosts, ranging from 1 : 6400 to 1 : 2,04,800, significantly greater than the control group mice given only PBS and adjuvant. Likewise, antibody titers of immunized group mice sera against the M1 peptide (1 : 1600 to 1 : 10,2400), the M12 peptide (1 : 800 to 1 : 51,200), and the J14 peptide (1 : 400 to 1 : 6400) are all higher than that of control group mice sera (*P* < 0.05, resp.) (Figure 2).

### 3.2. Bactericidal Activity of Antisera against Type 2 Streptococci

To test whether the antibody had the potential to kill M1 and M12 GAS, sera on day 55 were used to perform a bactericidal assay that measured GAS colony-forming units. As can be seen in Table 1, sera from immunized mice had significant level opsonic activity against these two type streptococci, resulting in 93.1% and 89.5% decrease of CFU, respectively.

### 3.3. Antibody Binds to the Surface of GAS

**Figure 3:**
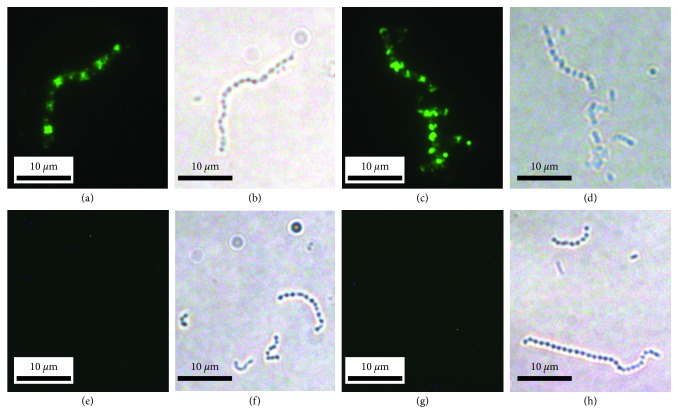
Photomicrographs of DyLight 488-stained M1 and M12 GAS. (a) M1 GAS exposed to antisera of immunized group mice. (b) Bright-field view of (a). (c) M12 GAS exposed to antisera of immunized group mice. (d) Bright-field view of (c). (e) M1 GAS exposed to antisera of control group mice. (f) Bright-field view of (e). (g) M12 GAS exposed to antisera of control group mice. (h) Bright-field view of (g).

Immunofluorescence was performed to visualize whether the antibody could connect to the streptococci. As Figure 3 shows, fluorescence appeared on the surface of emm1 and emm12 streptococci when antiserum of immunized mice were used as first antibody, while no fluorescence could be seen when antiserum of nonimmunized mice was used, which indicates that the divalent vaccine could elicit antibody that bound to the surface of the streptococci.

### 3.4. The Synthesized Divalent Vaccine Immunization Protects Mice against GAS Infection

**Figure 4:**
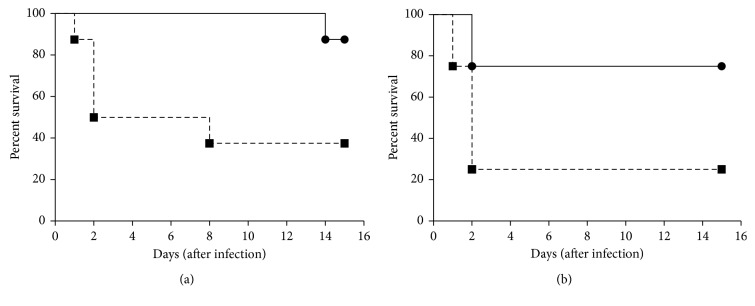
Protection of mice against group A streptococcus challenge after immunization with the vaccine. (a) Survival curves of mice challenged with M1 GAS. Immunized group mice (*n*=8, black circles) and control group mice (*n*=8, black squares) were intraperitoneally injected of 1.0 × 10^8^ CFU M1 GAS 21 days after the last immunization. Immunized group mice had a higher survival rate than nonimmunized group mice (87.5% versus 37.5%, *P*=0.0294). (b) Survival curves of mice challenged with M12 GAS. Immunized group mice (*n*=8, black circles) and control group mice (*n*=8, black squares) were intraperitoneally injected of 1.0 × 10^8^ CFU M12 GAS 21 days after the last immunization. Immunized group mice had a higher survival rate than nonimmunized group mice (75% versus 25%, *P*=0.0366). Statistical significance was determined by the log-rank test.

The synthesized divalent vaccine immunization elicited high titer antibody, and this antibody could bind to M1 and M12 streptococci and facilitate these streptococci to be killed, as described above. To test whether the antibody can protect mice, both immunized mice and nonimmunized mice were challenged with M1 and M12 streptococci, respectively. The immunized mice had 87.5% and 75% survival rate among 15 days, respectively (Figure 4), higher than that of nonimmunized mice (37.5% and 25%) (*P* < 0.05).

### 3.5. The Chemically Synthesized Divalent Vaccine Did Not Induce Autoimmune Reactions

**Figure 5:**
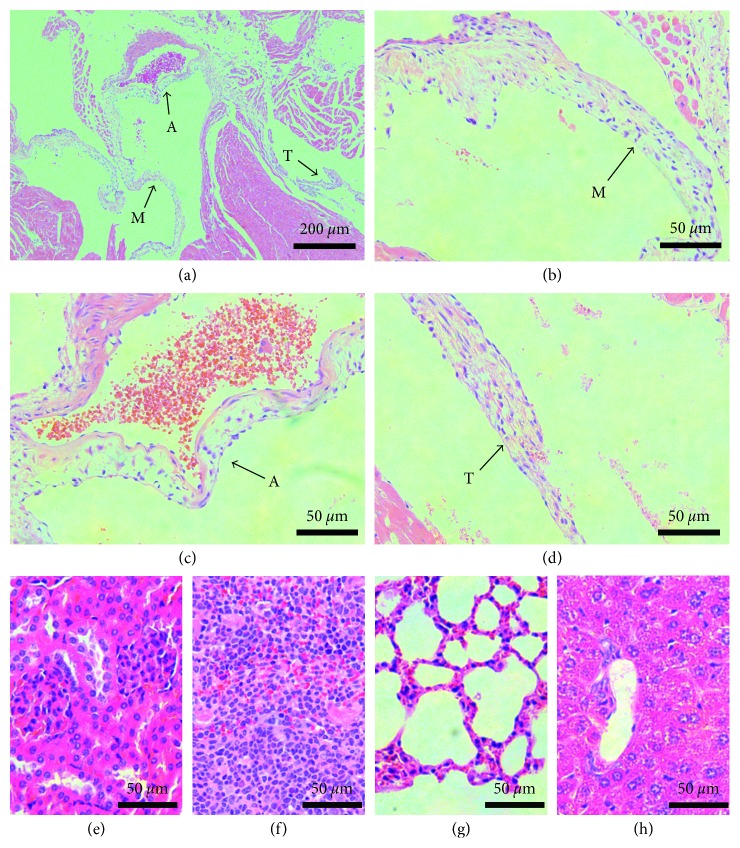
Histological analysis of tissues from mice immunized with high-dose peptide vaccine. Mice were immunized with 100 *μ*g peptide vaccine on days 1, 21, 31, and 41. Organs were collected 6 months after the last immunization. No autoimmune reactions were observed in these organs: (a) heart; (b) mitral valve; (c) aortic valve; (d) tricuspid valve; (e) kidney; (f) spleen; (g) lung; and (h) liver. M, mitral valve; A, aortic valve; T, tricuspid valve.

The safety of the synthesized divalent vaccine was examined by histological evaluation of tissues collected from mice 6 months after their last high-dose immunization. The results showed that no autoimmune reactions were observed in the heart and other organs (Figure 5).

## 4. Discussion

GAS is prevailing worldwide, and GAS-related diseases remain an important public health problem in both developed and developing countries. Vaccine is a feasible way to fight against the bacterium. Many research groups are trying to develop effective vaccines. Their strategies are based on different virulence factors, including GAS carbohydrate [18], serum opacity factor [19], GAS C5a peptidase [20, 21], and M protein. M protein is the major virulence factor of GAS, and currently research on M protein vaccine goes ahead of other virulence factors.

We developed the divalent vaccine based on M protein of emm1 and emm12 strains. Previously, we used molecular clone technology to synthesize the 84 amino acid peptide vaccine, while in this study, the peptide was synthesized by solid-phase peptide synthesis which is used by other research groups. We assessed the immunogenicity and bactericidal activity of the chemically synthesized peptide by ELISA assay and indirectly bactericidal assay, and this peptide shows similar immunological characteristics to the biosynthetic one used in our previous research. Then, we explored how the antibody is bactericidal against GAS by immunofluorescence assay, and it is demonstrated that the antibody is able to deposit on the surface of GAS, which is a potential reason for the bactericidal activity of the antibody.

The assays mentioned above were all in vitro experiments, and we also performed in vivo experiment to demonstrate that the vaccine was capable of protecting mice against GAS challenge. The result corresponded to our expectation because the indirect bactericidal assay had shown that the antibody promoted killing of GAS. One major concern about GAS vaccine based on M protein is the possibility of inducing autoimmune sequelae. Here, no autoimmune reactions and other pathological lesions were observed on mice receiving high-dose immunization, indicating that this vaccine is safe in mice. Thus, the questions raised on introduction were addressed; that is, this chemically synthesized divalent vaccine protects mice against GAS challenge and does not induce deleterious reaction. However, one limitation is that we could not get human tissue to test whether the antibody is cross reactive to human tissue, although cross reaction is of little possibility, because we had excluded five or more contiguous amino acids matching with human proteins when we designed the amino acid sequence of this divalent peptide vaccine.

We selected amino acid fragments from M protein of emm1 and emm12 GAS because these 2 serotypes are predominant strains in China according to several epidemiological surveys [11–14]. So this vaccine may prevent most streptococcal infection in our country. We note that there is a pursuit for a GAS vaccine effective in different regions of the world, but because of great variability of emm types in different regions and little cross reaction among heterogeneous serotypes, such a universally valid vaccine is unlikely to achieve. An alternative strategy is region-specific vaccine. As we know, N terminal of M protein has the highest immunogenicity. The dominant emm types of GAS in a given region can be determined through epidemiological investigation, and then safe and opsonic epitopes on N terminal of M protein of these dominant strains can be selected to compose a multivalent vaccine. Such a chemically synthesized multivalent vaccine is able to cover all the dominant strains and can be put into use in a short period of time. In underdeveloped areas such as Africa and the Pacific region where the existing GAS vaccines like the 26-valent vaccine, 30-valent vaccine, and StreptInCor may have a low coverage; a geographical region-specific multivalent vaccine is promising.

## 5. Conclusion

In summary, we have shown that this chemically synthesized divalent vaccine is immunogenic and safe in mice, and we believe that this strategy, developing a geographical region-specific multivalent vaccine, is worth extending to other areas.

## Figures and Tables

**Table 1:** Bactericidal activity of antisera to the divalent vaccine.

Serotype	CFU		Percentage reduction
	Immunized sera	Control sera	
M1	69	>1000	93.1
M12	105	>1000	89.5

CFU of one plate more than 1000 were counted as 1000. Percent reduction in CFU = (1 − (mean CFU from immunized sera/mean CFU from control sera)) × 100. Indirect bactericidal assays were performed by adding 50 *μ*L of M1 or M12 GAS to 50 *μ*L of immunized sera or control sera. Then, 400 of *μ*L nonopsonic heparinized human donor blood was added. The mixture was kept shaking for 3 h at room temperature, and then 50 *μ*L of mixture was plated on blood agar. CFU was quantitated after incubating overnight.
